# Refractory Chylous Ascites

**DOI:** 10.4021/gr2008.11.1252

**Published:** 2008-11-20

**Authors:** Rodrigo Soto, Ignacio García, Carlos Hinojosa, Aldo Torre

**Affiliations:** a Instituto Nacional de Ciencias Medicas y Nutricion “Salvador Zubiran” Vasco de Quiroga # 15, Col. Seccion XVI, Tlalpan, D. F. CP 14000, Mexico

**Keywords:** Chyloperitoneum, chylous ascitis, lymphangiectasias, lymphovenous shunt

## Abstract

A 34-year-old woman with primary chylous ascites due to lymphangiectasias was treated with sclerotherapy of dilated lymphatics and a lymphovenous shunt. She was referred to our institution after a thorough diagnostic and therapeutic approach in her community hospital. After four weeks of intensive diagnostic study, no secondary etiology for her chylous disorder was established. Conservative treatment did not prove useful, and a laparotomy was done. Lymphangiectasias and a lymphatic leak were demonstrated, but primary closure was ineffective. A second surgery with derivative intention was done, but six months later ascites recurred. A new sclerosing surgery was done; afterwards, the patient remained free of symptoms. Primary chyloperitoneum is a rare and complex disorder; its treatment and outcome depend on a multidisciplinary approach and an experienced medical team.

## Introduction

Chylous ascites is defined as the accumulation of triglyceride-rich, milk-like peritoneal fluid in the abdominal cavity. Most chylous disorders are rare and commonly due to secondary conditions. Once secondary causes are excluded, primary chylous disorders are often suspected. Primary chylous disorders represent a diagnostic and therapeutic challenge; they can be congenital or present later in life. In this article we report a refractory case of primary chyloperitoneum treated with sclerotherapy and a lymphovenous shunt.

## Case Report

A 34- year- old woman from the north of Mexico presented nonspecific abdominal complaints one month before she developed new-onset tense ascites. Her medical history included a hemithyroidectomy 12 years earlier due to a papillary thyroid cancer, an abdominal myomectomy 4 years ago, and a cesarean section done 4 months prior to admission to her community hospital. An abdominal paracentesis was done, revealing a milky fluid. Diagnostic study was extensive, but etiology for chylous ascites could not be established even after laparoscopic examination. One month later she was referred to our institution for diagnostic and treatment.

**Figure 1:**
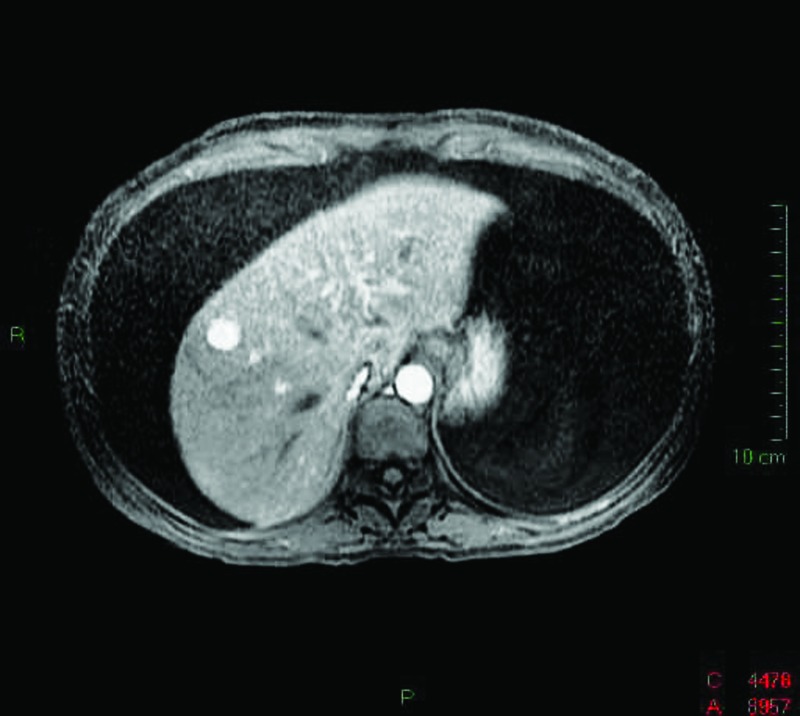
MRI showing massive ascites and a hepatic hamangioma.

**Figure 2:**
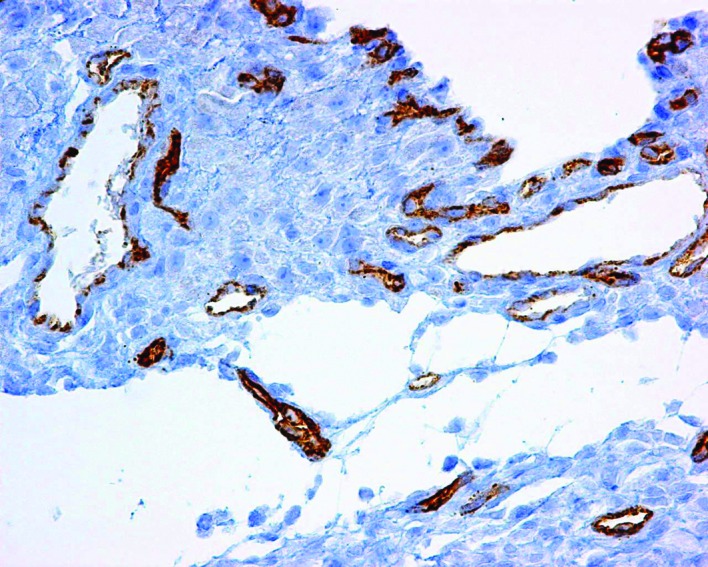
Peritoneum showing dilated lymphatics with normal CD34 positive endothelium.

Laboratory workup showed normal complete blood count (CBC), liver transaminases, and urinalysis; serum albumin of 1.8g/dL, ascites-triglyceride concentration of 2291mg/dL, serum-ascites albumin gradient (SAAG) <1.1g/dL, and negative ascitic cytology and cultures. Abdominal ultrasound (US), computed tomography (CT), and magnetic resonance (MRI) showed ascites and a hepatic hemangioma (Fig.1). Chest CT was normal, and the patient was negative for Mantoux test and adenosine deaminase (ADA) in ascitic fluid. Biopsy specimens from her referral hospital showed chronic peritonitis and dilated lymphatics (Fig.2). The patient was started on a low-fat diet supplemented with medium-chain triglycerides (MCT) and abdominal paracentesis as needed (10Lt/week).

Initial treatment did not prove useful; therefore, total parenteral nutrition (TPN) and octreotide acetate depot (20mg IM) were started. After a four-week period of intensive diagnostic study, no clear etiology for the chylous disorder was established. Lymphatic anatomy was evaluated with a whole-body lymphangioscintigraphy (LAS) that showed normal tracer distribution. Surgical exploration was planned to clarify the etiology and offer operative management.

**Figure 3:**
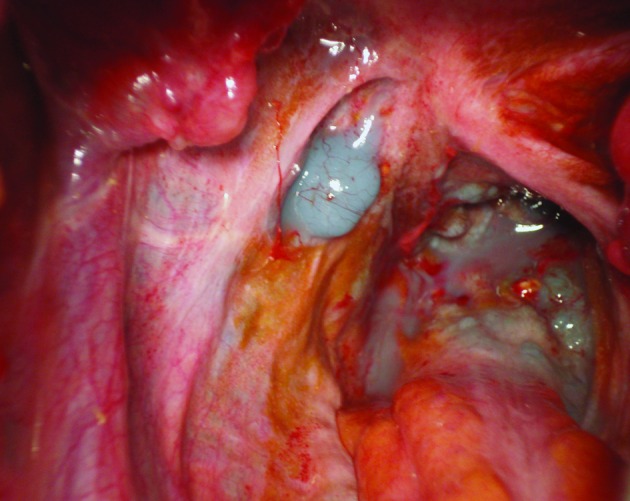
Intraoperative picture showing lymphangiectasia and a lymphatic leak (chylous effusion is stained with Sudan Black B).

Sudan Black B (5 grams) plus a fat-rich diet were administered eight hours before surgery. Laparoscopic examination did not prove useful, so it was converted to open exploration. Lymphangiectasias and a lymphatic leak from retroperitoneum were saw at rectouterine pouch (pouch of Douglas). Primary closure with sutures and fibrin glue was done (Fig.3). Fourteen days after surgery vaginal discharge was noted (200 ml/day). Colposcopic exploration showed copious cervical discharge similar to the ascitic fluid. A new surgical approach was planned.

Prior to resection of retroperitoneal, mesenteric, and pelvic lymphatics, sclerotherapy of dilated vessels was done. Iodine (60 ml) and sterile talc (4 grams) were diluted in 100 ml of normal saline and irrigated directly to provoke obstructive lymphangitis. A side to side lymphovenous shunt to the left ovarian vein was made from a large lymphatic channel (4 mm) located below the renal arteries. Postoperative evolution was favorable, but six months later the patient recurred with non-tense chylous ascites. A third surgery revealed thrombosis of the lymphovenous shunt, with no evidence of large lymphatic channels. Sclerotherapy with iodine and talc was performed again. The patient has been without evidence of ascites for eight months.

## Discussion

Chylous ascites is a rare disorder. At large reference centers, its incidence represents around one in 20,000 admissions over a 20-year period(1). The incidence is increasing, perhaps because of more aggressive retroperitoneal surgery treatments and longer survival for cancer patients(1, 2).

Diagnostic paracentesis is most important in any patient with ascites. The serum-ascites albumin gradient (SAAG) calculation is the first diagnostic approach and a useful portal pressure prognosticator(3). Chylous ascites is suspected by gross analysis, but definitive diagnosis is established by measuring ascites-triglyceride concentration, which is usually above 110-200 mg/dL(1-4).

After confirmation of a chylous disorder, most important step in clinical practice is to look for its etiology. In Western countries approximately 75% are malignancy or cirrhosis related. Lymphomas account for 33-50% of all cases; breast, colon, and ovarian cancer are also associated(1). Infectious diseases such as tuberculosis and filariasis are common causes in developing countries(2).

Treatment for chylous ascites starts with dietary modifications. High protein, low-fat and MCT supplementation successfully resolves lymphatic leaks in up to 50% of cases(4, 5). If no improvement is noticed, second line therapies (TPN, octreotide) should be started. Resolution rates for these therapies had been reported from 60 to 100%(4, 6).

First and second line therapies failed for this patient. LAS was performed in order to define lymphatic anatomy and the site of the lymphatic leak, but it did not prove useful. Even after a thorough investigation no signs of cancer, cirrhosis or infection were demonstrated. Less common causes of chylous ascites include congenital, inflammatory, traumatic, and postoperative disorders.

Postoperative chylous effusions result from injury to the thoracic duct, *cisterna chyli* or its intestinal tributaries. It can occur from a few days after surgery (lymphatic disruption) up to several months later (adhesions)(1). No case has been reported associated to cesarean section(7).

Next step in diagnosis and treatment is surgical exploration. Preparation included a fat-rich diet and a lipophilic dye to depict lymphatic anatomy(8, 9). Surgery main findings were a lymphatic leak and dilated vessels; therefore, the most probable diagnosis is a primary chylous disorder.

Primary chylous disorders are rare, and most of them are caused by congenital lymphangiectasia. They can be accompanied with obstruction, agenesis, hypoplasia, disruption or dysplasias(5, 8). Less frequent causes include the Yellow nail syndrome and Lymphangioleiomyomatosis(1, 2).

During the first surgery, primary closure of lymphatic leak was attempted(9). Success for this therapy has been described in about 80% of cases (8), but chylocolporrhea demonstrated that the problem persisted. A multidisciplinary team discussed the remaining therapeutic alternatives, and a second surgical approach was planned. New surgery objectives were sclerotherapy, resection of lymphangiectasic tissue, and a derivative resolution(10). Lymphovenous and peritoneovenous (Denver) shunts were both considered(10, 11), but lymphatic characteristics made feasible a lymphovenous shunt. Postoperative evolution was favorable, but after 6 months the problem recurred. A new surgery was done, no dilated lymphatics were observed and retroperitoneal sclerotherapy was performed. Eight months later, the patient is free of symptoms, but there is a great concern about a new recurrence.

Conclusions: Primary chylous disorders are rare and complex conditions that represent a diagnostic and therapeutic challenge. Almost half of these patients will require surgery, and their outcome significantly depend on the medical experience and technology available(5, 8, 10). Proper follow up is important, because one third of patients with primary chylous ascites will recur even when proper treatment has been instituted(5).
